# Etiology and clinical characteristics of a non-cystic fibrosis bronchiectasis cohort in a middle eastern population

**DOI:** 10.1186/s12890-023-02543-z

**Published:** 2023-07-10

**Authors:** Irfan Shafiq, Ali Saeed Wahla, Mateen Haider Uzbeck, Zaid Zoumot, Mohamed Abuzakouk, Shuayb Elkhalifa, Govinda Bodi, Khalaf Mohamed Almazrouei, Kashyap Bodi, Said Isse

**Affiliations:** Cleveland Clinic Abu Dhabi, Abu Dhabi, United Arab Emirates

**Keywords:** Bronchiectasis, Aetiology, Obstructive lung disease, Post-infectious

## Abstract

**Background:**

Bronchiectasis is a widely prevalent airway disease characterized by airway dilatation and recurrent infections, that can lead to respiratory failure in severe cases. The etiology of bronchiectasis varies geographically, but there is a lack of published data examining its etiology specifically within the Middle Eastern population.

**Methods:**

We conducted a retrospective analysis of our bronchiectasis patient registry, extracting clinical and demographic characteristics from electronic medical records. Quantitative variables were presented as the median and interquartile range (IQR), while categorical variables were expressed as numbers and percentages. Statistical comparisons for continuous characteristics were performed using the t-test, and significance was determined by a p-value less than 0.05.

**Results:**

In total we analysed 260 records (63% female, 37% male), with median age of 58 years (interquartile range (IQR) 38–71), Body Mass Index (BMI) 25.8(IQR 22–30), forced expiratory volume in the first second (FEV1) %predicted 65 (IQR 43–79) and FEV1/forced vital capacity (FVC) 0.76 (0.67–0.86). Sixty-five cases (25%) were post-infectious in aetiology (excluding post-TB – n:27 10.4%). Forty-eight (18.5%) patients were labelled idiopathic, while Primary Ciliary Dyskinesia (PCD) accounted for 23 (8.8%) cases. Pseudomonas aeruginosa was the most common colonizing organism (32.7%), followed by Haemophilus influenzae (9.2%) and Methicillin-Sensitive Staphylococcus aureus(6.9%). At the time of review, 11 patients had died (median age, FEV %predicted, and bronchiectasis severity index (BSI) 59 years, 38% and 15.5 respectively), all due to respiratory failure, and as expected, all were classed severe on BSI. The BSI score was available for 109 patients, of which 31(28%) were classed mild, 29(27%) were moderate, and 49 (45%) were classed severe. The median BSI score was 8 (IQR 4–11). On dividing the patients according to obstructive vs. restrictive spirometry, we found that patients with FEV1/FVC < 0.70 had significantly higher BSI (10.1 vs. 6.9, p-value < 0.001) and that 8 out of the 11 deceased patients had FEV1/FVC < 70%.

**Conclusions:**

In our study, post-infectious, idiopathic, and PCD were identified as the most common etiologies of bronchiectasis. Additionally, patients with obstructive spirometry appeared to have a worse prognosis compared to those with restrictive spirometry.

## Introduction

**Fig. 1:**
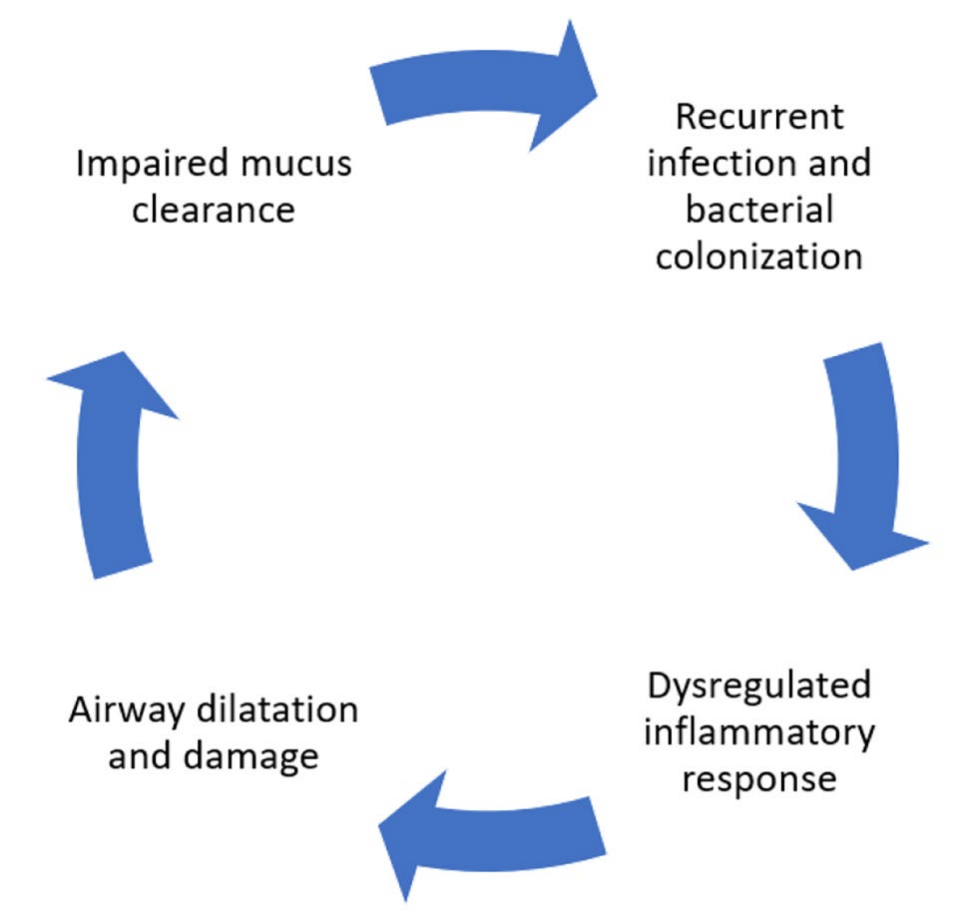
Cole’s vicious cycle hypothesis

Bronchiectasis is a common chronic lung disease characterized by recurrent infections due to defective mucus clearance from the airways and can progress to chronic respiratory failure and death. Bronchiectasis can either be idiopathic or secondary to another disease process, and finding the etiological cause can alter management and, in some cases, alter the prognosis [1]. The pathogenesis is usually explained through Cole’s model, which delineates the vicious cycle of airway damage, leading to impaired mucus clearance, resulting in chronic infection and bacterial colonization that continues to drive the inflammatory process and leads to further airway damage (Fig. 1) [2]. Significant geographic differences exist in the etiology of bronchiectasis worldwide [3]. The investigations for the etiological causes can be expensive and knowing the prevalence of various etiological causes in a population can help to plan the investigations and reduce the cost. Previously published data has shown significant heterogeneity in the investigations performed at various centers and also in definitions of etiological causes [4–8] however, a recent study has proposed standardized algorithm based diagnosis strategy to help overcome the variations in epidemiology [9]. The European Respiratory Society (ERS) published guidelines for bronchiectasis management in 2017 which recommends a minimum bundle of investigations that includes complete blood count (CBC), Immunoglobulin A (IgA), Immunoglobulin M (IgM) and immunoglobulin G (IgG) in all bronchiectasis patients [1] with further investigations to be done on the clinical suspicion and on the treating physician’s discretion. Recent research has shown that further immunological testing is usually needed to identify more patients with treatable immunodeficiencies such as primary or secondary antibody deficiencies [10] and doing so can alter the course of bronchiectasis for those patients.

To date there are no published data on the etiology of bronchiectasis in the Middle East and in this study, we review the etiology along with demographic and clinical parameters of bronchiectasis in the local population in the United Arab Emirates.

## Methods

### Population

**Fig. 2:**
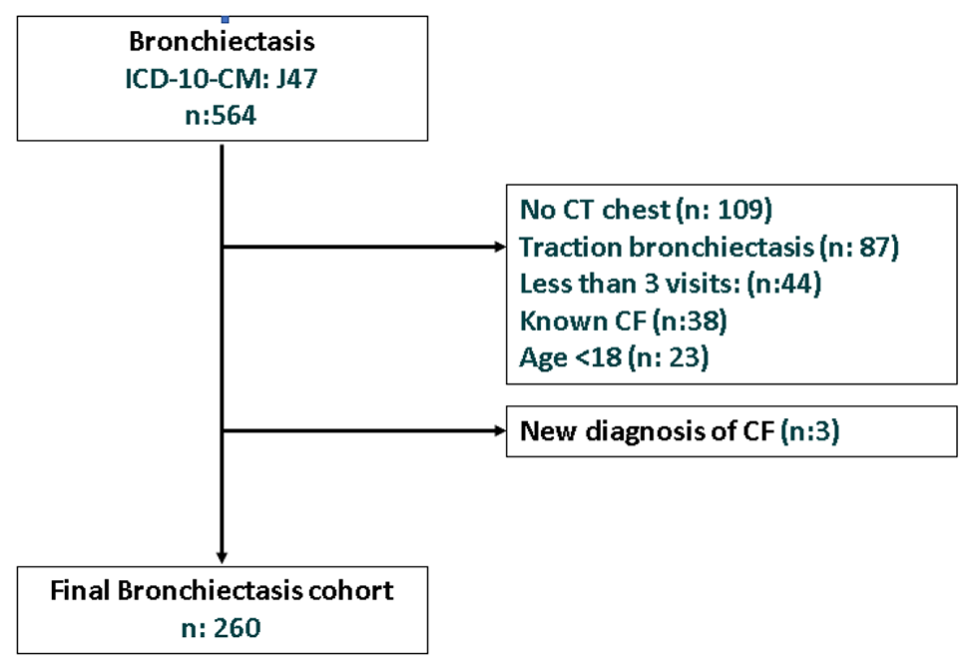
Bronchiectasis cohort flow diagram, 2014 to 2021

The Electronic Health Records (EHR) at our tertiary care hospital were searched with the diagnosis code bronchiectasis (ICD-10-CM: J47) between April 2014 and December 2021. We included the patients who had the diagnosis confirmed on the basis of a high-resolution computerized tomography (CT) chest and at least 3 visits to the Pulmonary clinic. Patients under the age of 18, or with known cystic fibrosis (CF) or diagnosed with CF during the investigation were excluded from final analysis (Fig. 2). We identified a total of 260 patients that met the inclusion/exclusion criteria. Local Research Ethics Committee approval was obtained and the research methods were carried out in accordance with declaration of Helsinki.

### Study variables

Basic demographic data was collected for all study participants along with clinical data including spirometric values, etiological investigations for primary or secondary immunodeficiencies and for hereditary conditions such as CF and primary ciliary dyskinesia (PCD). Sweat chloride was used to diagnose CF, and in patients with positive or indeterminate sweat test or those with high clinical suspicion for it, CF mutation testing was done initially using a 97-mutation panel and, if negative, then through gene sequencing. Nasal nitric oxide and PICADAR score were used to screen patients for PCD. In addition, screen-positive patients had respiratory mucosal brushings sent for transmission electron microscopy. Patients with typical Kartagener’s syndrome and those who already had siblings with an established diagnosis of PCD did not have any of the above investigations. The sputum culture results and the scores of the bronchiectasis severity index (BSI) were also recorded. If all investigations were non-diagnostic, aetiology was labelled “post-infectious” based on the past medical history of pulmonary TB, pertussis, measles or severe pneumonia in childhood. Without the past history of infections, aetiology was labelled “idiopathic”.

### Statistical analysis

Quantitative variables are expressed as the means and standard deviation if normally distributed or median and interquartile range (IQR) if otherwise. Categorical variables are expressed as numbers and percentages [11]. Relationship between various study variables was assessed using Pearson’s correlation coefficient. Statistical comparisons between continuous characteristics were carried out using the two tailed t-test, and a significant p-value was taken to be less than 0.05. Data analysis was done with MS Excel 2019.

## Results

Two hundred and sixty of 561 patient records reviewed (63% female, 37% male) met the inclusion criteria and were included in the final analysis (Fig. 2). The median age of the patients was 58 years (IQR 38–71), median BMI 25.8(IQR 22–30), FEV1% predicted was 65 (IQR 43–79) and FEV1/FVC 0.76 (0.67–0.86). The baseline characteristics of the cohort are described in Table 1.

**Table 1:** Baseline characteristics

	n	Median	(IQR)
Age	260	58	(38–71)
BMI	260	25.8	(22–30)
FEV1%	226	65	(43–79)
FVC%	226	69	(50–82)
FEV1/FVC	226	76	(67–86)
IgG	177	1185	(970–1471)
IgM	177	0.85	(0.63–1.3)
IgA	177	2.98	(2.1–3.9)
Eosinophil count (10^9^/L)	260	0.28	(0.18–0.48)
Total IgE	214	82	(27–347)
BSI score	109	8	(4–11)
BSI severity	109	2	(1–3)

**Fig. 3:**
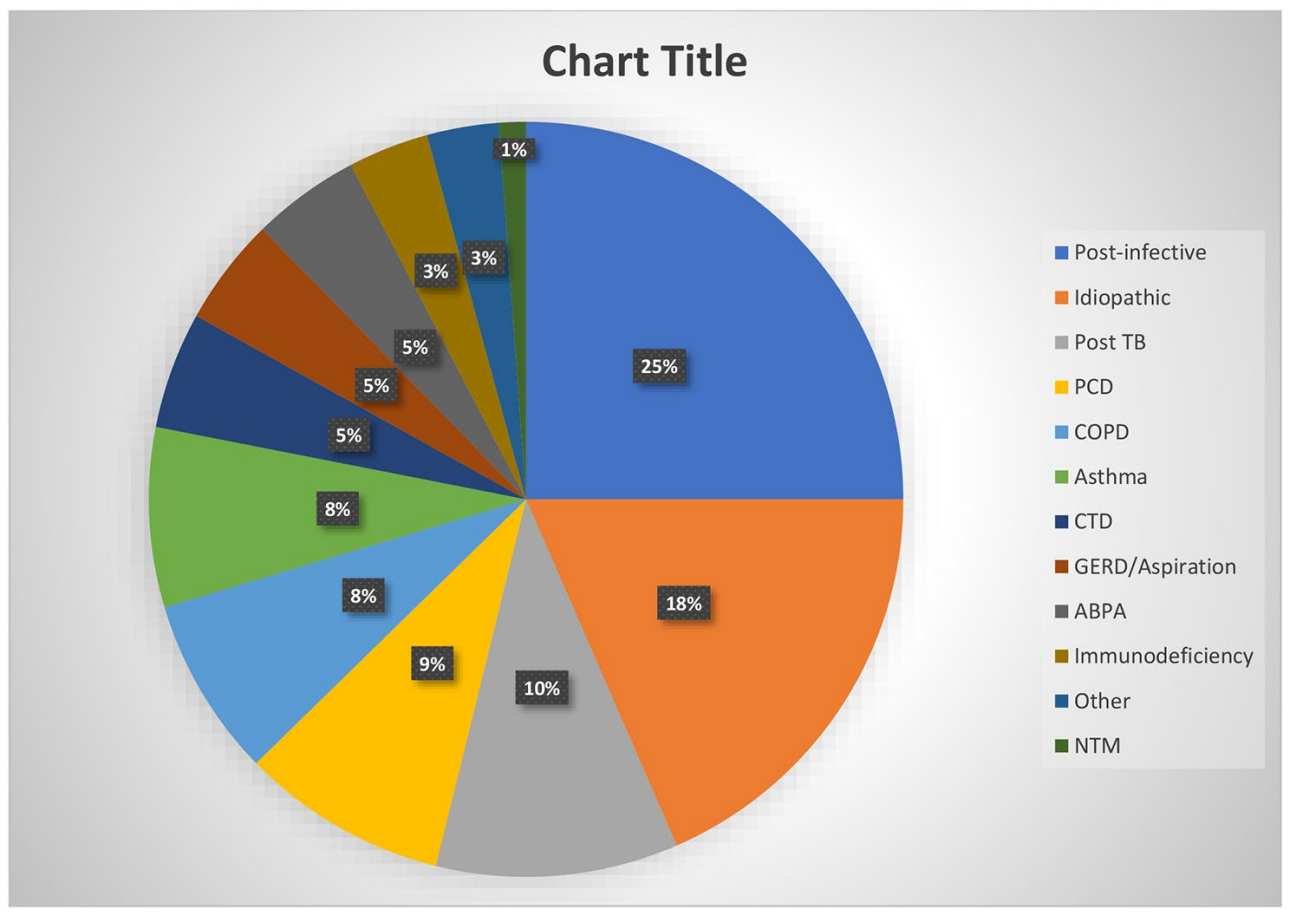
Bronchiectasis etiologies

A total of 65 cases (25%) were post-infectious in aetiology (excluding post-tuberculosis, n:27–10.4%). Forty-eight (18.5%) patients were labelled idiopathic, while PCD accounted for 23 (8.8%) of cases. Nine (3.5%) cases were due to underlying immunodeficiencies, these included primary and secondary immunodeficiencies as follows: Two cases of Common Variable Immunodeficiencies (CVID), one case of Chronic Granulomatous disease (CGD), and six cases of antibody deficiencies. The complete breakdown of aetiology is shown in Table 2; Fig. 3.

**Table 2:** Etiology

Etiology	n	%
Post-infective	65	25.0
Idiopathic	48	18.5
Post TB	27	10.4
PCD	23	8.8
COPD	20	7.7
Asthma	20	7.7
CTD	13	5.0
GERD/Aspiration	12	4.6
ABPA	12	4.6
Immunodeficiency	9	3.5
Other	8	3.1
NTM	3	1.2

Pseudomonas aeruginosa (32.7%) was the most common colonizing organism (Table 3), followed by Haemophilus influenzae (9.2%), Methicillin-Sensitive Staphylococcus aureus (MSSA) (6.9%) and Methicillin-resistant Staphylococcus aureus (MRSA) (3.8%). BSI was available for 109 patients, of which 31(28%) were classed mild, 29(27%) were moderate, and 49 (45%) were classed severe. The median bronchiectasis severity index (BSI) score was 8 (IQR 4–11). At the time of review, 11 patients were deceased (median age FEV% and BSI 59,38 and15.5 respectively), all due to respiratory failure and all were classed severe on BSI.

**Table 3:** Clinical Characteristics

Column1	n	%
**Total**	260	
Deceased	11	4.2
**Bacterial colonization**		
Pseudomonas	85	32.7
MSSA	18	6.9
MRSA	10	3.8
H influenzae	24	9.2
Stenotrophomonas	3	1.2
NTM	3	1.2
**Long-term antibiotics**		
Azithromycin	116	44.6
Inhaled Tobramycin	18	6.9
Inhaled Colistin	20	7.7

On dividing the patients according to obstructive vs. restrictive spirometry, we found that patients with FEV1/FVC < 0.70 had significantly higher mean BSI (10.1 vs. 6.9, p < 0.001) and that eight out of the 11 deceased patients had FEV1/FVC < 0.70. We also found that patients colonized with pseudomonas had significantly lower mean FEV1%predicted when compared to those without pseudomonas colonization (55.6 vs. 61.1, p < 0.001). The patients with peripheral eosinophilia (defined as serum eosinophil count > 0.3 10^9^/L) also had significantly higher mean BSI than those without eosinophilia however this difference did not correlate with FEV1% predicted (Table 4).

**Table 4:** Comparitive charecteristics of bronchiectasis cohort

Parameters			n	mean	median	Std Dev	p-value
BSI (points)	FEV1/FVC	< 70%	70	10.1	11	4.71	< 0.001
		> 70%	39	6.9	7	4.70	
	Eosinophilia	present	15	11.2	11	4.4	0.007
		absent	94	7.5	7	4.85	
FEV1 (% predicted)	Pseudomonas colonization	present	77	55.6	57.5	22.38	< 0.001
		absent	146	66.1	69	21.66	
	Eosinophilia	present	34	57.9	61	23.54	0.13
		absent	193	63.7	66	22.12	

Pearson’s correlation coefficient was used to look for relationship between FEV1%predicted and various demographic and clinical parameters including age, BMI, Immunoglobulins levels and number of hospital visits, but no statistically significant positive or negative correlations were detected.

## Discussion

Our study investigated the clinical characteristics and aetiology of non-CF bronchiectasis in a cohort of patients followed up in a tertiary care hospital in the Middle East. There was a predominance of female patients in the studied cohort, which is in keeping with most of the published data [12]. The median age of our cohort was 58 years of age. It is noteworthy that several previous studies have shown increasing prevalence of bronchiectasis in older adults [13, 14]. The median age of general population in Abu Dhabi is only 32 years [15] which is significancy younger than most developed countries. It is thus likely that as the population gradually ages, the prevalence of bronchiectasis will also increase. The BSI is a validated clinical prognostication tool used to risk-stratify patients with bronchiectasis. It takes into account age, BMI, hospitalization history, exacerbation frequency, spirometry, sputum colonization, MRC dyspnea score and radiological severity. The BSI assigns points to each parameter and classifies the severity into different categories. BSI helps guide treatment decisions and provides insights into future exacerbations and mortality risks, with a higher BSI score being associated with a increased rate of hospitalization and mortality [14]. In our study, the calculated BSI was mild in 28%, moderate in 27% and severe in 45% of the cohort. Age is an important prognostic factor in patients with bronchiectasis with older patients having more symptoms and hence, age is one of the components that make up the BSI [14] however we were not did not find a significant correlation between age and FEV1 in our patient group.

The predominant spirometry pattern in our cohort was obstructive which was also associated with worse clinical outcome. We found patients with obstructive spirometry had significantly higher mean BSI (10.1 vs. 6.9) compared to patients with restrictive spirometry. In addition, eight out of the 11 deceased patients in our cohort had obstructive spirometry. These findings are compatible with what is described in the literature previously [16]. Patients with peripheral eosinophilia seemed to have higher disease severity as judged by BSI in our study. This may be because most ABPA patients (8 out of 12) had peripheral eosinophilia. Of other obstructive airway disease patients, only 3 asthmatics and 3 COPD patients demonstrated peripheral eosinophilia.

There are well documented geographic variations in the prevalence of various aetiologies of bronchiectasis [3]. In our study, the most common aetiology was post infectious (excluding post TB). When added together, post infectious and post tuberculosis cases accounted for 35% of patients. The second commonest aetiology in our cohort were those labelled as idiopathic, while PCD was the fourth most common cause found in our cohort. The post-infectious and idiopathic aetiologies are common in Asian patients as shown in a recent study from Taiwan which found post infectious and in particular post-tuberculosis as the most common aetiology [17]. However, PCD as the third most common aetiology in our cohort is somewhat unusual and needs more explanation. In a previous French study of inherited factors for diffuse bronchiectasis in adults, PCD was determined as the aetiology in 13% of the study population, however that cohort only had 38 patients with diffuse bronchiectasis, with 5 patients having a diagnosis of PCD. Four out of the 5 patients with PCD diagnosis in this study were of African descent [18]. A paediatric study from India (n:80) reported suspected PCD in 15% of study participants [19]. Most other larger studies of bronchiectasis have shown much lower prevalence of PCD [3] and it is possible to deduce from available data that prevalence of PCD may be higher in the non-Caucasian populations. This observation is further supported by the results from our cohort. Furthermore, the higher prevalence of PCD in our study may be explained by several other factors which include a higher proportion of younger patients, high rates of consanguinity in the region and the fact that our hospital is the main referral centre in the country for complex bronchiectasis patients.

In our study Pseudomonas aeruginosa (32%), Hemophilus influenzae (9%) and MSSA (7%) were the pathogens most commonly isolated. Only three patients had NTM (1.2%) colonization in sputum. Previous studies have reported major geographic variations in microbiology [3] but pseudomonas predominance in our cohort is in keeping with prior published data. The incidence of TB and NTM are known to be inversely related [20], probably due to cross-immunity, and high endemicity of TB in UAE probably accounts for low NTM rates in our study population.

Bronchiectasis is a common chronic lung disease and is the main cause of mortality and morbidity in patients with primary or secondary antibody-deficiencies [21, 22]. It is also the most commonly reported post-infectious complication in both paediatric-onset and adult‐onset common variable immunodeficiencies [23]. However, immunodeficiency is a treatable cause of bronchiectasis if diagnosed and treated early with immunoglobulin replacement therapies (either intravenously or subcutaneously). Patients with bronchiectasis and immunodeficiency may benefit from a higher dose of immunoglobulin replacement because immunoglobulin therapy may reduce airways inflammation and improve the mucous expectoration [21]. In our cases of immunodeficiencies and bronchiectasis, we have identified three definite primary immunodeficiencies (CVIDs & CGD) with early onset disease and six cases with variable causes of antibody deficiencies presented as an adult-onset disease.

The current ERS guidelines only recommend measurement of complete blood count along with IgG, IgA and IgM as the baseline set of investigation for immunodeficiency in bronchiectasis [1]. Of course, with nonspecific signs/symptoms of immunodeficiencies, the more extensive investigations are likely to yield greater number of patients with a diagnosis albeit a much higher health-economic burden. In a recent study comparing five different sets of immunological investigations, Aliberti et al. were able to demonstrate that addition of IgG subtypes and lymphocyte subsets to the base investigations recommended in ERS guidelines resulted in a very significant increase in immunodeficiency diagnoses (8.9% vs. 44.6% p < 0.001) and further addition of HIV test and IgE did not increase the diagnostic yield any further [10]. IgG subsets were tested for in about 24% of patients in our cohort while none of the patients had lymphocyte subsets tested which may partly account for lower immunodeficiency diagnosis in this study.

One obvious limitation of our study is the retrospective design which of course reduces the quality of the data and makes it difficult to exclude confounders. However, to date all the studies looking at the bronchiectasis aetiology have been of retrospective observational nature and that could at least partly explain the heterogeneity of results. The lack of standardization of investigations especially the ones focused on immunodeficiency testing has also been a significant problem and probably leads to underestimation of this immunodeficiency disorders. The recent data form Aliberti et al. is likely to address this issue in near future [10].

In conclusion our study is the first one from the Middle Eastern region that shows aetiological factors in the regional population of Arab ethnicity. The data shows similarities to other western cohorts with high number of post-infective and idiopathic cases with high rate of pseudomonas colonization but contrasts in terms of higher post-TB and PCD related cases.

## Data Availability

The datasets used and/or analyzed during the current study available from the corresponding author on reasonable request.
